# A novel therapeutic approach for inflammatory bowel disease by exosomes derived from human umbilical cord mesenchymal stem cells to repair intestinal barrier via TSG-6

**DOI:** 10.1186/s13287-021-02404-8

**Published:** 2021-05-29

**Authors:** Shaopeng Yang, Xiaonan Liang, Jia Song, Chenyang Li, Airu Liu, Yuxin Luo, Heran Ma, Yi Tan, Xiaolan Zhang

**Affiliations:** 1 Department of Gastroenterology, The Second Hospital of Hebei Medical University, No. 80 Huanghe Road, Yuhua District, Shijiazhuang, 050000 Hebei China; 2 Shandong Qilu Cell Therapy Engineering Technology Co., Ltd, Jinan, Shandong China

**Keywords:** Inflammatory bowel disease, Mesenchymal stem cells, Exosome, Tumor necrosis factor-α-stimulated gene 6, Intestinal barrier

## Abstract

**Background:**

Exosomes as the main therapeutic vectors of mesenchymal stem cells (MSC) for inflammatory bowel disease (IBD) treatment and its mechanism remain unexplored. Tumor necrosis factor-α stimulated gene 6 (TSG-6) is a glycoprotein secreted by MSC with the capacities of tissue repair and immune regulation. This study aimed to explore whether TSG-6 is a potential molecular target of exosomes derived from MSCs (MSCs-Exo) exerting its therapeutic effect against colon inflammation and repairing mucosal tissue.

**Methods:**

Two separate dextran sulfate sodium (DSS) and 2,4,6-trinitrobenzenesulfonic acid (TNBS)-induced IBD mouse models were intraperitoneally administered MSCs-Exo extracted from human umbilical cord MSC (hUC-MSC) culture supernatant. Effects of MSCs-Exo on intestinal inflammation, colon barrier function, and proportion of T cells were investigated. We explored the effects of MSCs-Exo on the intestinal barrier and immune response with TSG-6 knockdown. Moreover, recombinant human TSG-6 (rhTSG-6) was administered exogenously and colon inflammation severity in mice was evaluated.

**Results:**

Intraperitoneal injection of MSCs-Exo significantly ameliorated IBD symptoms and reduced mortality rate. The protective effect of MSCs-Exo on intestinal barrier was demonstrated evidenced by the loss of goblet cells and intestinal mucosa permeability, thereby improving the destruction of tight junctions (TJ) structures and microvilli, as well as increasing the expression of TJ proteins. Microarray analysis revealed that MSCs-Exo administration downregulated the level of pro-inflammatory cytokines and upregulated the anti-inflammatory cytokine in colon tissue. MSCs-Exo also modulated the response of Th2 and Th17 cells in the mesenteric lymph nodes (MLN). Reversely, knockdown of TSG-6 abrogated the therapeutic effect of MSCs-Exo on mucosal barrier maintenance and immune regulation, whereas rhTSG-6 administration showed similar efficacy to that of MSCs-Exo.

**Conclusions:**

Our findings suggested that MSCs-Exo protected against IBD through restoring mucosal barrier repair and intestinal immune homeostasis via TSG-6 in mice.

## Introduction

Inflammatory bowel disease (IBD) is a chronic and non-specific inflammatory gastrointestinal disease, with ulcerative colitis (UC) and Crohn’s disease (CD) as the common subtypes of IBD. IBD is mainly caused by inappropriate immune response of genetically susceptible hosts to pathogens [1], and its characteristics include abnormal mucosal immune response and intestinal barrier function disorder [2]. With the rising incidence of IBD, existing therapies cannot meet the clinical patients’ needs [3]. Accordingly, the urgent development of safe and effective treatments against IBD is necessary.

Mesenchymal stem cells (MSC) are multipotent progenitors with differentiating capabilities, which can be isolated from different tissues, such as adipose, umbilical cord, and bone marrow [4]. MSC transplantation has been considered as a novel therapeutic approach for IBD with the potential to regulate immune response and promote tissue regeneration [5]. Previous study has shown that less than 1% of MSC injected intravenously homing at the damaged intestinal tissue [6]. In addition, intraperitoneal injection of conditioned media (CM) from MSCs is shown to alleviate the symptoms of experimental colitis and reduce the levels of TNF-α and MMP2 in mice [7]. These studies strongly support the beneficial effects of MSC as an attribute to the paracrine pathway. Considering the stem cell transplantation shortcomings, such as cell rejection, high cost, and potential risk of malignant transformation [8], exosomes secreted by MSCs (MSCs-Exo) have attracted widespread attention.

Exosomes are 40-160 nm bilayer membrane vesicles that mediate cell-to-cell communication and paracrine factor transportation [9]. Current studies reported that MSCs-Exo exert similar immune regulation and tissue repair properties as stem cells in many autoimmune diseases [10–12]. The therapeutic effects of MSCs-Exo have been demonstrated in colitis mouse model [13–15]. In terms of mechanisms, studies recently reported that MSCs-Exo attenuate colitis through increasing the proportion of Treg cells and M2 macrophages [16, 17]. However, the effect of MSCs-Exo on the intestinal mucosal barrier remains unclear.

Tumor necrosis factor-α stimulated gene 6 (TSG-6) is a 30-kDa immunomodulatory molecule secreted by MSC or immune cells during inflammation irritation [18]. Yang et al. found that human-induced pluripotent stem cell (iPSC)-derived MSC could promote epithelial cell proliferation and accelerate mucosal repair through TSG-6 in a colitis mouse model [19]. However, whether MSCs-Exo-secreted TSG-6 plays a critical role in intestinal barrier maintenance is still unknown. In this study, we examined the therapeutic effect of MSCs-Exo in IBD treatment and revealed that MSCs-Exo repair the mucosal barrier and maintain the balance between Th2 and Th17 cells mainly through TSG-6. These findings provide novel insights into the mechanism of MSCs-Exo-mediated intestinal repair, thus contributing to the development of cell-free IBD therapy.

## Materials and methods

### hUC-MSC isolation and culture

Human umbilical cord-derived MSC (hUC-MSC) were provided by Shandong Qilu cell therapy Engineering Technology Co., Ltd. hUC-MSC isolation was performed in the Current Good Manufacturing Practice (cGMP)-accredited laboratory. After obtaining written informed consent, human umbilical cords (hUC) were harvested. Wharton’s jelly was minced into 1-mm^3^ small pieces and cultured in mesenchymal stem cells basic medium (Beijing Yocon Biology Co., Ltd.) supplemented with a free-serum replacement. Migration of primary cells from the tissue was about 7 days.

Flow cytometry was performed to examine the expression of cell surface markers for hUC-MSC characterization. Positive cell surface markers CD90, CD105, CD73, CD44, and negative surface markers of CD34, CD45, and HLA-DR were characterized. Meanwhile, multilineage differentiation of adipogenesis, osteogenesis, and chondrogenesis were conducted using a commercially available differentiation kit to evaluate the multilineage differentiation capacity of hUC-MSC.

### Characterization of exosomes

hUC-MSC were cultured without serum for 48 h and cell culture supernatant was collected. Culture supernatant was centrifuged to remove dead cells and cell debris, as described in the previous study [20]. After centrifugation, culture supernatant was filtered with a 0.22-μM pore filter (Merck KGaA, Darmstadt, Germany). The filtered supernatant was concentrated using a 150-kD Protein Concentrator (Millipore, Massachusetts, USA) and filtered again with a 0.22-μM pore filter. Exosomes were isolated from the final filtered supernatant with ExoQuick-TC exosome isolation reagent (System Biosciences, California, USA) according to the manufacturer’s protocol. Finally, the precipitated exosomes were resuspended in sterile phosphate-buffered saline (PBS) and stored at − 80 °C.

Characterization of extracted exosomes was performed by transmission electron microscopy (TEM) to observe the morphology. Nanoparticle tracking analysis (NTA) was conducted to analyze the particle size and video image of exosomes. Western blot was performed to detect two exosome surface markers (TSG101, CD9, Calnexin, and CD63).

### Small interfering RNA (siRNA) transfection

hUC-MSC were thawed and plated in 24-well plates in serum-free medium. TSG-6 (siTSG-6) and negative control (siNC) siRNAs were purchased from Suzhou Genepharma Co. (Suzhou, China). hUC-MSC were transfected with TSG-6 or negative control siRNAs using Lipofectamine 2000 (Invitrogen, Carlsbad, CA, USA) when hUC-MSC reached approximately 80% confluence to obtain TSG-6 knockdown MSCs. The culture medium was changed 6 h after the hUC-MSC transfection. After 24 h, the culture supernatant was collected to extract TSG-6 knockdown exosomes (siTSG-6 Exo) and negative control exosomes (siNC Exo). To verify transfection efficiency of TSG-6, RNA, and protein were extracted from TSG-6 siRNA and negative control siRNA-transfected MSC, followed by quantitative PCR (qPCR) and western blot analysis. Levels of TSG-6 in exosomes and supernatants with exosome depletion were detected by enzyme-linked immunosorbent assay (ELISA). Before performing ELISA, the protein concentration of all samples was unified.

### IBD mouse models

All animal experiments were approved by the Local Animal Ethics Committee. Specific pathogen-free male C57BL/6 mice aged 6–8 weeks (weighing 18–22 g) and BALB/C mice aged 7–8 weeks (weighing 20–22 g) were purchased from Beijing Vital River Laboratory Animal Technology Co. Ltd. Two mouse models for colitis were induced separately by oral gavage of dextran sodium sulfate (DSS) (MP Biomedicals, USA) and rectal infusion of 2, 4, 6-trinitrobenzenesulfonic acid solution (TNBS) (Sigma-Aldrich, USA) according to that previously described [21].

In TNBS-induced acute colitis model, 1% TNBS (1.5 mg/mouse) pre-sensitization solution was applied to the skin on the back of male BALB/c mice. After 7 days from pre-sensitization step, 100 μl of 2.5% TNBS (2.5 mg/mouse) solution was slowly injected into the anus of anaesthetized mice. Mice injected with 50% ethanol without TNBS were used as control (control group, n = 5). Exosome treatment was performed via intraperitoneal injection of 200 μg exosomes per mouse after 24 h of rectal administration (TNBS+Exo group, n = 5). PBS was intraperitoneally injected into mice as control (TNBS+PBS group, n = 5).

DSS-induced acute colitis was induced in male C57BL/6 mice by oral administration of 2% DSS in drinking water for 7 days, and control mice received water without DSS (control group, n = 8). In total, 200 μg of MSCs-Exo (DSS + Exo group, n = 16), siTSG-6 MSCs-Exo (DSS + siTSG-6 Exo group, n = 8), or siNC MSCs-Exo (DSS + siNC Exo group, n = 8) was injected intraperitoneally 5 days after DSS treatment. To determine the efficacy of TSG-6 in DSS-induced colitis model, recombinant human TSG-6 protein (rhTSG-6) (R&D, USA) was injected daily at 4 μg/mouse (DSS + TSG-6 group, n = 5) starting from day 5 to day 9 as described in previous studies [6]. Control group received PBS (DSS + PBS group, n = 6) administration at the same time.

### Myeloperoxidase (MPO) activity assay

Infiltration of neutrophils into colonic tissue was quantified by MPO activity measurement with an MPO assay kit (Nanjing Jiancheng Bio-engineering Institute, Nanjing, China) according to the manufacturer’s instructions. MPO activity was expressed as units per gram of total protein (U/g). The localization and expression of MPO were detected by immunohistochemical staining (see “Immunohistochemical analysis” for detailed methods).

### Assessment of colitis

Mice were monitored daily and assessed for disease severity using the numerical system of DAI as described previously [21]. Colon tissue samples were fixed in formaldehyde and stained with hematoxylin and eosin (H&E). Histopathological damage analysis of DSS colitis model was determined to measure the severity of inflammation using Cooper HS score system [22]. As for TNBS colitis model, another scoring system was used to evaluate inflammation-associated histological changes according to the previous study [21]. Periodic Acid-Schiff (PAS) staining was performed using PAS dye solution set (Servicebio, Wuhan, China) to evaluate the structure of colonic goblet cells. Quantitative analysis of purple-red goblet cells was evaluated using ImageJ software.

### Intestinal permeability assay

Intestinal mucosal permeability was evaluated by feeding the mice with fluorescein isothiocyanate-dextran (FITC-D, 4 kDa; Sigma-Aldrich, St. Louis, USA) on the 10th day after DSS administration. The mice were fasted for 4 h and lavaged with FITC-D (60 mg/100 g) before sacrifice. The serum was collected by centrifugation at 12,000*g* for 5 min, and the level of FITC-D in the serum was detected using a fluorescence spectrophotometer (excitation wavelength = 490 nm, emission wavelength = 520 nm). The intestinal tissue was embedded in OCT and sectioned (5 μm thick). The sections were stained with DAPI (blue), and then the distribution of FITC-D (green) in the intestinal was observed using a fluorescence microscope.

### Western blot

RIPA lysis buffer was used (Solarbio, Beijing, China) to extract protein in the colon tissue and exosomes. Protein concentrations were determined by BCA Protein Assay Kit (Solarbio, Beijing, China). Equal aliquots of protein (30 μg per lane) were separated by 10% SDS polyacrylamide gel and transferred to polyvinylidene fluoride (PVDF) membranes (Millipore Corp, Billerica, MA, USA). The PVDF membranes were blocked with 5% milk for 1 h, followed by incubation with primary antibodies against Claudin-1 (Abcam, Cambridge, MA), Occludin (Abcam, Cambridge, MA), ZO-1 (Abcam, Cambridge, MA), and TSG-6 (Affinity Biosciences, OH, USA) at 4 °C overnight. The protein bands were visualized and analyzed using the Odyssey CLx imaging systems (Li-COR Biosciences, Lincoln, NE, USA).

### Quantitative PCR

The mRNA levels of TSG-6 in colon tissue were determined using quantitative PCR (qPCR). Total RNA in colon tissue was isolated by TRIzol (Ambion, Carlsbad, CA, USA), and the RNA concentration and quality were assessed by spectrophotometric analysis. The purified RNA was reversely transcribed using FastKing RT kit (Tiangen, Ltd., Beijing, China) according to the manufacturer’s protocol and preserved at − 20 °C. The qPCR was performed to measure the transcript abundance of the genes with Synergy Brands (SYBR) Green detection (Applied Biosystems, Carlsbad, CA). Expression levels of TSG-6 were normalized with GAPDH expression level to calculate relative expression values. Primer sequences of TSG-6 were as follows: 5′-GGGATTCAAGAACGGGATCTTT-3′ (forward), 5′-TCAAATTCACAT ACGGCCTTGG-3′ (reverse).

### Flow cytometry analysis

Splenic and mesenteric lymph node (MLN) cells were isolated as previously described [23]. For intracellular cytokine staining, the cells were incubated with Cell Activation Cocktail (Biolegend, San Diego, USA) in 5% CO_2_ at 37 °C for 6 h. After stimulation, the cells were stained with anti-CD4 (Biolegend, San Diego, USA) and incubated with anti-interferon (IFN)-γ, anti-IL-17A antibodies, or anti-IL-4 for 1 h at 4 °C. For flow cytometry analysis, cells were acquired on a FACSVerse flow cytometer (BD Biosciences, San Jose, CA) and analyzed using FlowJo software (TreeStar, USA).

### Immunohistochemical analysis

The expression and distribution of the tight junction proteins in colon tissue were analyzed by immunohistochemical staining. Mice colonic tissue were fixed in 4% paraformaldehyde and embedded in paraffin. The paraffin-embedded tissue were cut into 4-μm sections for immunostaining based on the procedures described in the previous study [24]. The following primary antibodies were used: Claudin-1 (Abcam, Cambridge, MA), Occludin (Abcam, Cambridge, MA), ZO-1 (Abcam, Cambridge, MA), and MPO (Abcam, Cambridge, MA). Images were taken under a light microscope at ×400 magnification, and expression of tight junction protein in epithelial cells was analyzed using ImageJ as described in the previous study [25].

### Cytokine gene microarray

Gene expression profiles were analyzed using mouse Cytokines and Chemokines qPCR Array according to the manufacturer’s protocol (Wcgene Biotech, Shanghai, China). Data were analyzed using Wcgene Biotech software. Genes with fold-changes more or less than 2.0 were considered to be biological significant.

### Transmission electron microscopy

The colon tissues were fixed with fresh TEM fixative (Servicebio, Wuhan, China) at 4 °C and washed with PBS (pH 7.4). Tissues were dehydrated at room temperature using ethanol and embedded in resin. Resin blocks were sectioned into 60–80 nm thin and stained with uranium acetate and lead citrate for 8 min. Images were taken under a transmission electron microscope (H7800; Hitachi Ltd., Japan).

### Enzyme-linked immunosorbent assay (ELISA)

All samples were adjusted to the same protein concentration before testing. Levels of TSG-6 in exosomes or cell culture supernatant with exosomes were detected by the ELISA kit (CUSABIO, Wuhan, China) according to the manufacturer’s instructions. The concentrations of TSG-6 were calculated according to standard curve generated by the Curve Expert software.

### Statistical analysis

Data were presented as the mean ± standard deviation (SD). Multiple groups were compared using one-way analysis of variance (ANOVA) followed by Tukey’s multiple comparison tests. Unpaired Student’s *t*-test was used to compare the differences between two groups. Mouse survival curves were calculated using the Kaplan-Meier method, and the *p* value was determined by a log-rank test possibly adjusted for comparison between two groups. *P*<0.05 was considered as statistically significant. Statistical analysis was performed using GraphPad Prism version 8.0 for Windows (GraphPad Software, San Diego, CA, USA).

## Results

### Isolation and characterization of MSCs-Exo

**Fig. 1:**
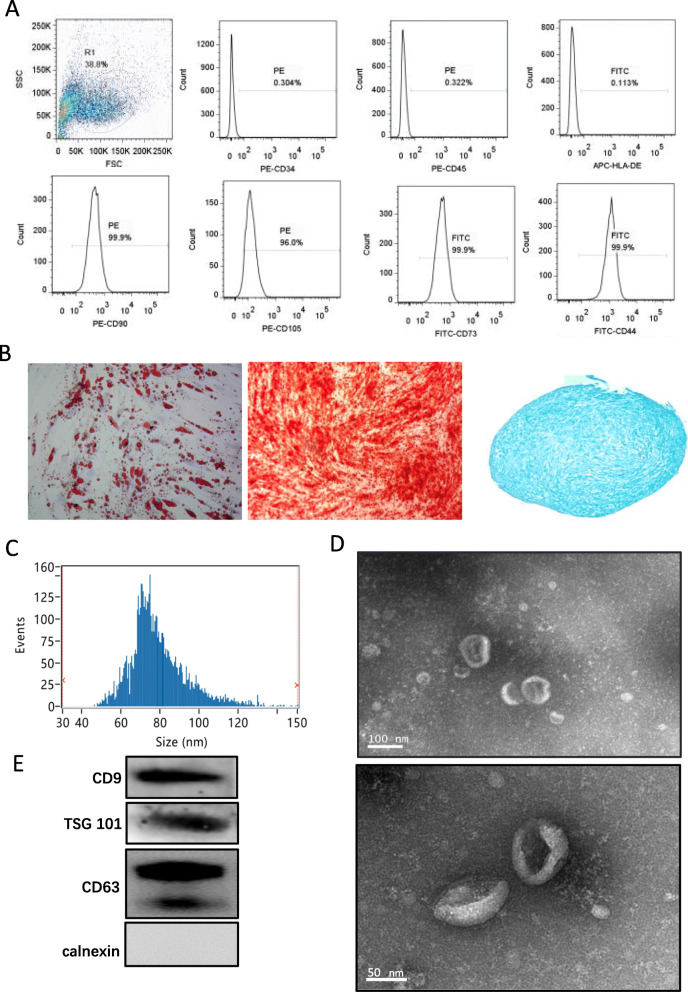
Characterization of hUC-MSC and MSCs-Exo. **a** hUC-MSC were characterized by flow cytometry for detection of positive cell surface markers (CD44, CD73, CD90, and CD105) and negative markers (CD34, CD45, and HLA-DR). **b** The multilineage differentiation of hUC-MSC into adipocytes (Nil oil red O staining), osteoblasts (Alizarin red S staining), and chondrocytes (Alcian blue staining). The particle size distribution profile of exosomes was indicated in **c** by NTA. **d** TEM images showing the morphology of exosomes. Upper scale bar: 100 nm; bottom scale bar: 50 nm. **e** Western blot analysis showing the protein expression of CD9, CD63, TSG 101, and calnexin

hUC-MSC were isolated from the human umbilical cord and the expression of cell surface markers were analyzed using flow cytometry. Results revealed that the positive surface markers of hUC-MSC were CD44, CD73, CD90, and CD105, while the negative markers were CD34, CD45, and HLA-DR (Fig. 1a). hUC-MSC were able to differentiate into osteogenic, adipogenic, and chondrogenic lineages (Fig. 1b). The particle size distribution profile of exosomes were indicated by NTA (Fig. 1c). The diameters of MSCs-Exo were in the range of 30–150 nm. The size and morphology of MSCs-Exo were observed under transmission electron microscopy (TEM). Results showed that MSCs-Exo appeared as circular discs, representing a normal morphology of MSCs-Exo (Fig. 1d). Western blot analysis showed the expression of MSCs-Exo markers, including CD9, CD63, and TSG101, while calnexin, a negative marker, was not detected (Fig. 1e). These results indicated successful isolation of exosomes from hUC-MSC culture supernatant.

### MSCs-Exo attenuated chemical-induced colitis in mice

**Fig. 2:**
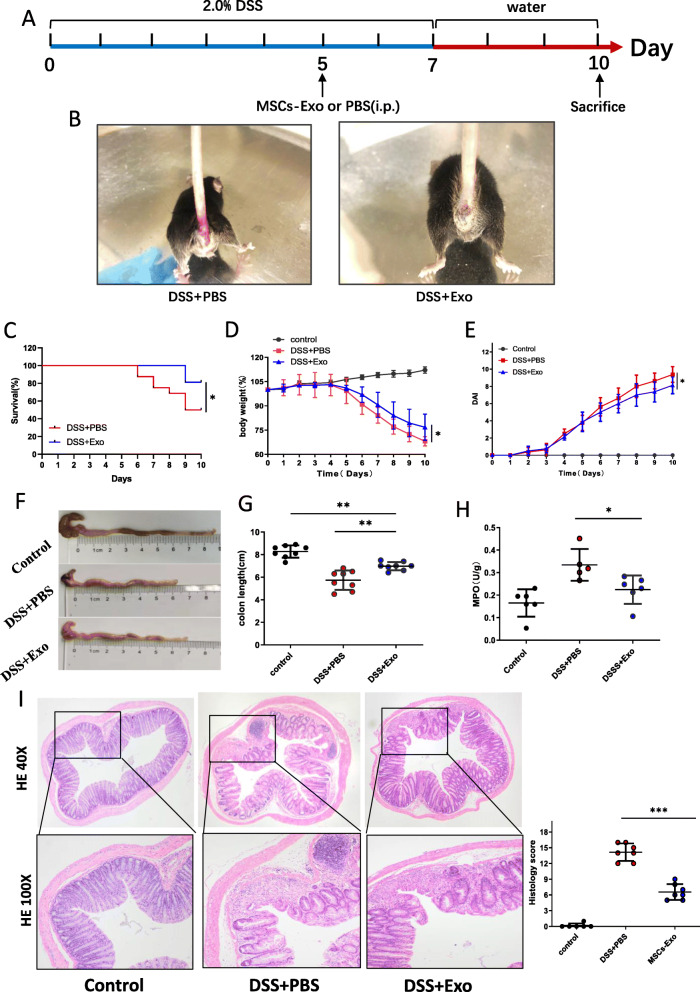
MSCs-Exo attenuated DSS-induced acute colitis. **a** Schematic diagram of the experimental design. Mice were administered with 2.0% DSS continuously for 7 days to induce colitis. Mice were intraperitoneally injected with the same volume of exosomes or PBS at day 5, and sacrificed at day 10. i.p., intraperitoneal. **b** Hematochezia in the DSS + PBS mice was more serious than mice of the DSS + Exo group at day 7. **c** Survival rate of mice in the DSS + PBS and DSS + Exo groups (n = 16). The body weight (**d**) and DAI (**e**) of mice were recorded daily from day 0 to day 10. Colonic macroscopic images (**f**), colon length (**g**), MPO (**h**), HE staining and histological score (**i**) in the control, DSS + PBS, and DSS + Exo groups at day 10. Data are presented as mean ± SD. *P < 0.05, **P < 0.01, and ***P < 0.001

**Fig. 3:**
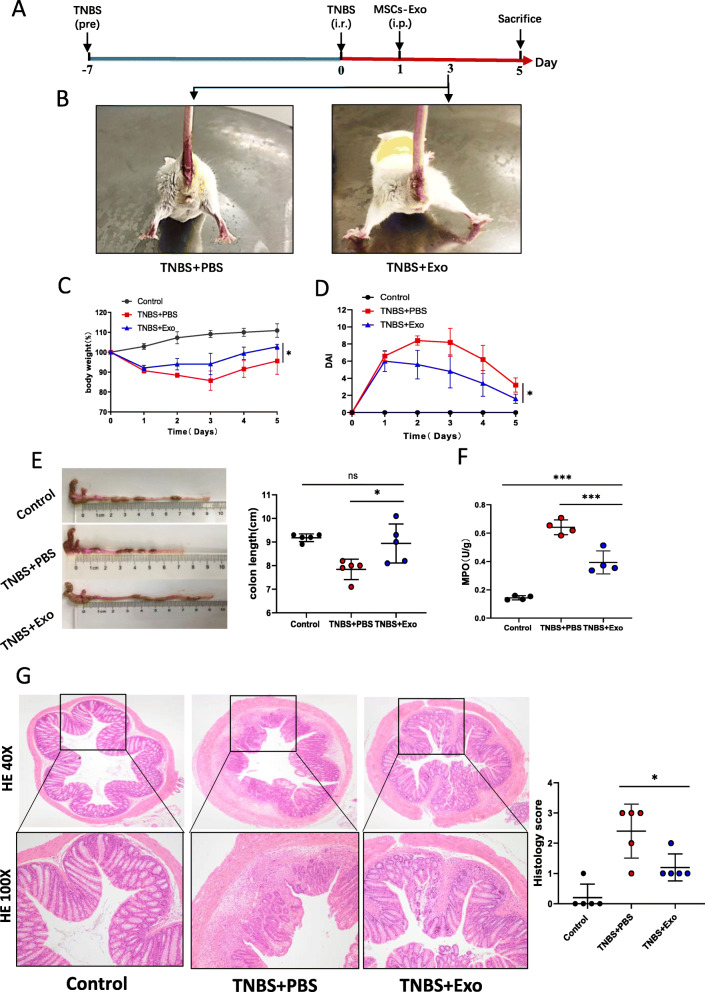
MSCs-Exo alleviated TNBS-induced acute colitis. **a** Schematic diagram of the experimental design. Mice were intraperitoneally injected with MSCs-Exo 1 day after enema of TNBS, and mice were sacrificed 5 days after TNBS treatment. Pre: before treatment; i.r., intrarectal delivery; i.p., intraperitoneal. **b** Severe diarrhea and bloody stool in the TNBS+PBS mice at day 3. The body weight (**c**) and DAI (**d**) of each group of mice were recorded daily (n = 5 per group). **e** Gross morphology and length of colonic tissues in the control, TNBS+PBS, and TNBS+Exo groups at day 5. **f** The infiltration of neutrophils into colon was quantified by the MPO activity. **g** Histopathological changes were evaluated by hematoxylin and eosin (H&E) staining of colon tissue and histological score. Original magnification, × 40 (upper), × 100 (lower). Data are presented as mean ± SD. *P < 0.05; **P < 0.01; ***P < 0.001, and ns indicates *P* > 0.05

Therapeutic effects of MSCs-Exo on IBD were explored through DSS- and TNBS-induced colitis. In DSS-induced colitis, the survival rate of mice treated with MSCs-Exo (DSS + Exo group) was significantly higher than mice treated with PBS (DSS + PBS group) on day 10 (Fig. 2c). Moreover, the DSS + PBS group exhibited lower body weight and increasing DAI scores compared with the DSS + Exo group (Fig. 2d, e). On the 10th day of the treatment, the colonic length of the DSS + PBS group was shorter than that in the DSS + Exo group (Fig. 2f, g). MPO activity reflects the infiltration of neutrophils in the colon tissue. As shown in Fig. 2h and Fig.S4a, the MPO activity and MPO-positive cells were significantly elevated after oral administration of DSS, whereas MSCs-Exo-treated mice exhibited no increase in MPO activity and MPO-positive cells. Histological scoring revealed that MSCs-Exo reduced the structural destruction of colon tissue, inhibited inflammatory cell infiltration, and crypt loss (Fig. 2i). The effect of MSCs-Exo against colitis was not limited to DSS-induced colitis. MSCs-Exo was also observed to significantly retard the progression of TNBS-induced colitis (Fig. 3b–g).

### MSCs-Exo alleviated intestinal mucosal barrier dysfunction in colitis mice

**Fig. 4:**
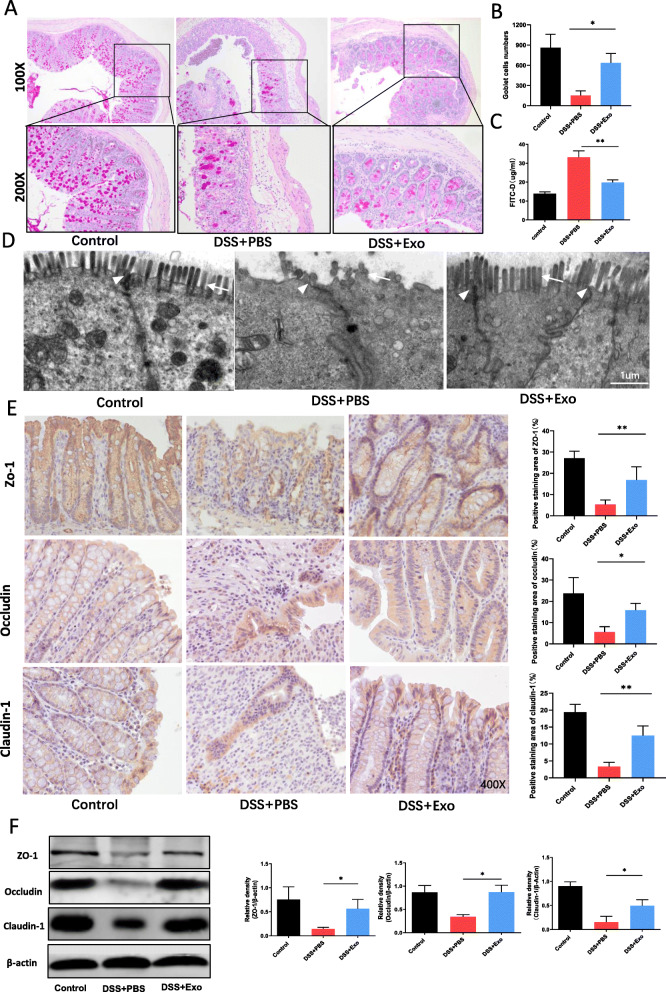
MSCs-Exo alleviated intestinal mucosal barrier dysfunction in colitis. **a** PAS staining showing the number of goblet cells (purple-red) in the colon. Original magnification, × 100 (upper), × 200 (lower). **b** Quantitative analysis showing the number of goblet cells. **c** Concentration of FITC-D in serum showing the permeability of the intestinal mucosa in FITC-D-treated mice. The ultrastructural morphology of tight junctions (white arrows) and microvilli (white arrowheads) were observed under TEM. Decreased tight junction electron density and loose microvilli reflecting the characteristics of intestinal mucosal barrier destruction. Scale bar, 1 μm. **e** Immunohistochemistry showing the expression levels of tight junction proteins ZO-1, Occludin, and Claudin-1 in the intestinal epithelium. Images were taken at × 400 magnification. **f** Quantitative analysis of the expression levels of the tight junction markers ZO-1, Occludin, and Claudin-1 in colonic mucosa by western blot. Data are presented as mean ± SD. *P < 0.05 and **P < 0.01

We used PAS staining to determine the number of goblet cells in colon, which were purple-red in color under a microscope. As shown in Fig. 4a and b, the number of colonic goblet cells in the DSS + Exo group was more than that in the DSS + PBS group. The concentration of FITC-D in serum was quantified to determine the intestinal barrier permeability after DSS administration. Result demonstrated that the permeability was significantly higher in DSS + PBS group than that in DSS + Exo group (Fig. 4c). Immunofluorescence co-localization showed that FITC-D was concentrated in the intestinal lumen of control group, and most of FITC-D penetrated into the intestinal mucosa in the DSS + PBS group. FITC-D penetration into the intestinal mucosa was reduced after MSCs-Exo treatment (Fig.S5). The ultrastructural morphology of tight junctions (TJs; white arrows) and microvilli (white arrowheads) were observed under TEM. Damaged TJs and loose microvilli were shown in the DSS + PBS group, representing the characteristics of intestinal mucosal barrier destruction. Conversely, the damaged TJ structure and microvilli were improved in the DSS + Exo group (Fig. 4d). The land expression of TJ protein ZO-1, Occludin, and claudin-1 in intestinal epithelial cells were determined by immunohistochemical staining and western blot. As shown in Fig. 4e and f, both immunohistochemistry and western blot results showed significantly higher protein expressions of ZO-1, Occludin, and Claudin-1 in the DSS + Exo mice compared to the DSS + PBS mice. We also performed similar studies in the TNBS-induced colitis mouse model and found that MSCs-Exo also exhibited a protective effect on the intestinal mucosal barrier (Fig. S3a-e and Fig.S4b).

### MSCs-Exo inhibited pro-inflammatory cytokine expression and promoted the expression of anti-inflammatory cytokines in DSS-induced colitis

**Fig. 5:**
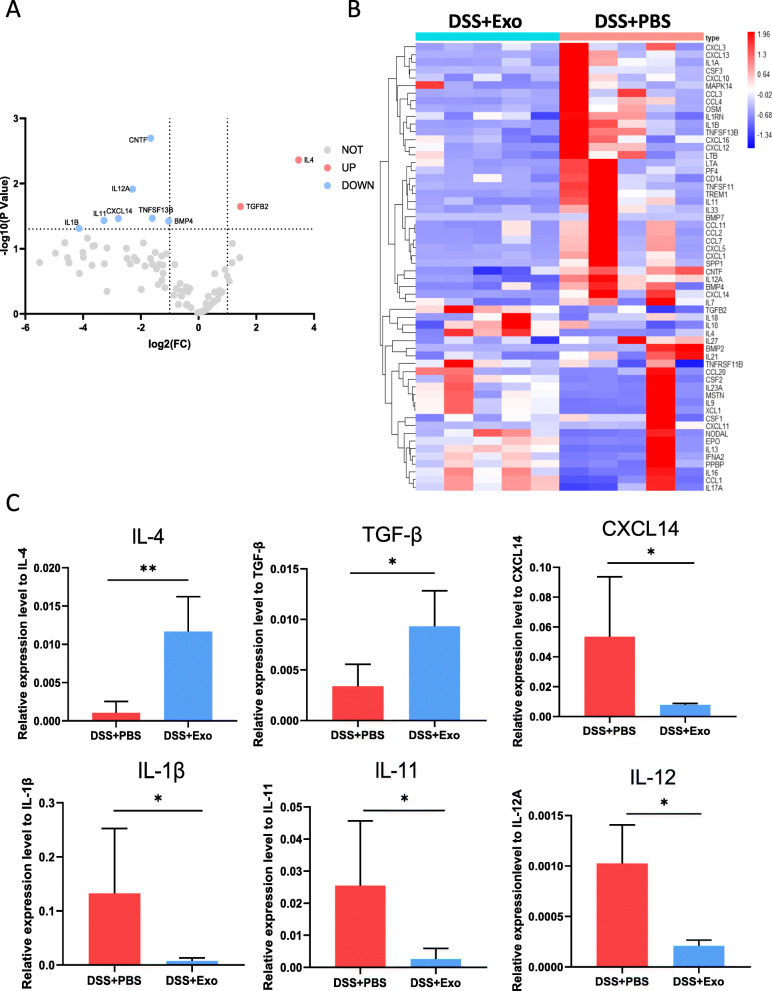
Treatment of MSCs-Exo altered cytokine profile in colon tissue. **a** Volcano plot showing differential expression of multiple mRNAs from colon tissue in the DSS + Exo group compared with the DSS + PBS group. **b** The hierarchical clustering representing cytokine mRNA at significantly different levels between the DSS + Exo group and the DSS + PBS group (fold-change>2, *P* < 0.05). In the heatmap, high expression is shown in red, and low expression is shown in blue. **c** qPCR results showing the mRNA levels of IL-4, TGF-β, CXCL14, IL-1β, IL-11, and IL-12 in the two groups. n = 5 per group. Data are presented as mean ± SD. *P < 0.05 and **P < 0.01

With the anti-inflammatory effects of MSCs-Exo demonstrated in previous results, the changes of cytokine expression profiles in colonic mucosal were also analyzed through cytokines and chemokines qPCR array following MSCs-Exo treatment. Volcano plot showed differential expressions in multiple mRNAs in the DSS + Exo group compared to the DSS + PBS group (Fig. 5a). The heatmap represented the hierarchical clustering of differentially expressed cytokine genes of the two groups (Fig. 5b). In the heatmap, high expression is shown in red, and low expression is shown in blue. qPCR results demonstrated the upregulation of pro-inflammatory factors in the DSS + PBS group, such as chemokine ligand 14 (CXCL14), Interleukin-1β (IL-1β), IL-11, and IL-12. Compared with the DSS + Exo group, anti-inflammatory factors IL-4 and TGF-β were downregulated in the DSS + PBS group (Fig. 5c).

### MSCs-Exo regulated the differentiation of CD4^+^ T cells in DSS-induced colitis

**Fig. 6:**
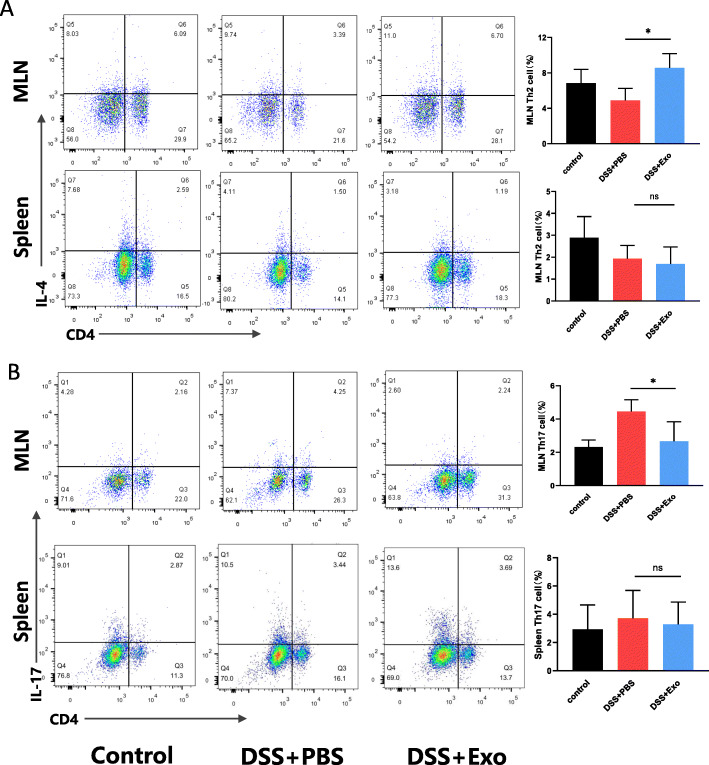
MSCs-Exo treatment altered the proportion of Th2 cells and Th17 cells in MLN of colitis mice. **a** Flow cytometry analysis showing the proportion of CD4^+^ IL-4^+^ (Th2) T cells in mesenteric lymph nodes (MLN) and spleen. The histogram represented the percentage of Th2 cells. **b** Percentage of cells expressing CD4^+^ IL-17^+^ (Th17) T cells from MLN and spleen. The histogram represented the percentage of Th17 cells. n = 4–5 per group, data are presented as mean ± SD. *P < 0.05 and ns indicates *P* > 0.05

Imbalance of T cell subsets plays an important role in the onset and progression of IBD. To explore the impact of MSCs-Exo administration on T helper cell differentiation, mononuclear cells were isolated from spleen and mesenteric lymph nodes (MLN) and analyzed by flow cytometry (Fig. 6, Fig. S2). The proportion of CD4^+^ IL-4^+^ (Th2) cells in MLN was significantly higher in the MSCs-Exo-treated mice compared with the untreated mice (Fig. 6a). In addition, the proportion of CD4^+^ IL-17A^+^ (Th17) cells in MLN was significantly lower in the MSCs-Exo-treated and control group mice compared with the untreated MSCs-Exo mice (Fig. 6b). However, the proportion of Th2 and Th17 cells in the spleen was not significantly different between the DSS + PBS and DSS + Exo groups (Fig. 6a, b). These data indicated that MSCs-Exo improved colitis by regulating the balance between Th2 and Th17 cells in MLN.

### MSCs-Exo enhanced TSG-6 expression in colon tissue with colitis

**Fig. 7:**
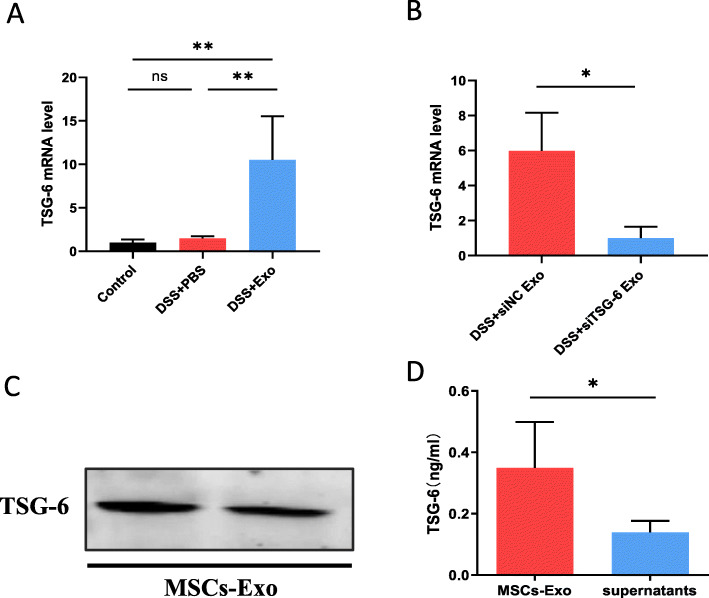
MSCs-Exo enhanced TSG-6 expression in the colitis mice colon. **a** TSG-6 expression levels in colon tissue in the control, DSS + PBS, and DSS + Exo groups 10 days after colitis induction. **b** TSG-6 expression in the DSS + siNC Exo and DSS + siTSG-6 Exo groups. **c** TSG-6 protein expression in MSC-Exo. **d** The levels of TSG-6 in MSC-Exo and supernatant without exosomes. Exosomes in the supernatant were removed by ultrafiltration. All samples were adjusted to the same protein concentration, followed by ELISA. Data are presented as mean ± SD; n = 4 per group. *P < 0.05, **P < 0.01 and ns indicates *P* > 0.05

TSG-6 is a immunomodulatory molecule secreted by MSCs and has tissue-protective properties. To explore which factors in MSCs-Exo play a role in ameliorating colitis, the expression level of TSG-6, a protective regulator against inflammation, was measured in colonic mucosa. The expression of TSG-6 mRNA in colitis mice with MSCs-Exo treatment was significantly higher than that of the DSS + PBS and control group (Fig. 7a). With siRNA-mediated knockdown of TSG-6, MSCs-Exo was unable to increase the expression of TSG-6 in the colonic mucosa from colitis mice (Fig. 7b). In addition, TSG-6 expression in the MSCs-Exo was also detected by western blot (Fig. 7c). ELISA demonstrated that the concentration of TSG-6 in MSCs-Exo was higher than that of hUC-MSC culture supernatant with exosome deletion (Fig. 7d). Collectively, the results suggested a potential role of TSG-6 in MSCs-Exo in colitis mice.

### Downregulation of TSG-6 reduced therapeutic effects of MSCs-Exo in alleviating colitis and protecting the intestinal barrier

**Fig. 8:**
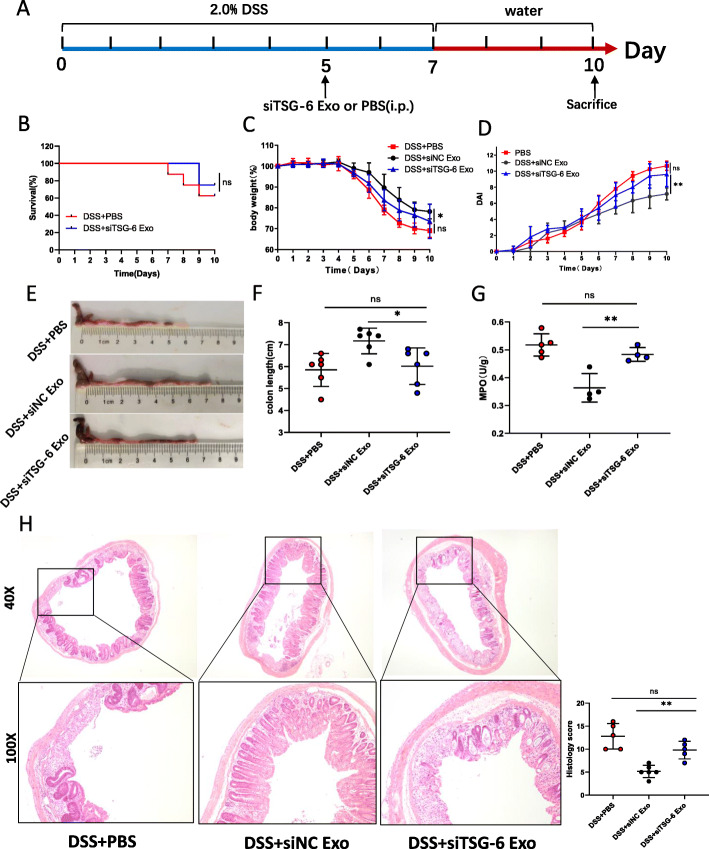
Downregulation of TSG-6 blocked the effect of MSCs-Exo in alleviating colitis. **a** Schematic diagram of the experimental design. **b** Survival rate of mice in the DSS + PBS and DSS + siTSG-6 Exo groups (n = 8 per group). Body weight (**c**) and DAI (**d**) were recorded daily during the experiment. Colonic macroscopic images (**e**), colon length (**f**), MPO (**g**), H&E staining, and histological score (**h**) in the DSS + PBS, DSS + siNC Exo, and DSS + siTSG-6 Exo groups at day 10. Data are presented as mean ± SD. *P < 0.05, **P < 0.01 and ns indicates *P* > 0.05

To investigate whether the effect of MSCs-Exo in the treatment of colitis was mediated by TSG-6, we knocked down TSG-6 in hUC-MSCs with siRNA targeting TSG-6 (Fig. S1a-c) and isolated exosomes (siTSG-6 Exo). There was no significant difference in the survival rate between the colitis mice treated with PBS and siTSG-6 Exo (Fig. 8b). The therapeutic effects of MSCs-Exo were weakened after knocking down TSG-6, which were manifested in body weight, DAI, histological score, colon length, and MPO activity (Fig. 8c–h and Fig.S4c).

**Fig. 9:**
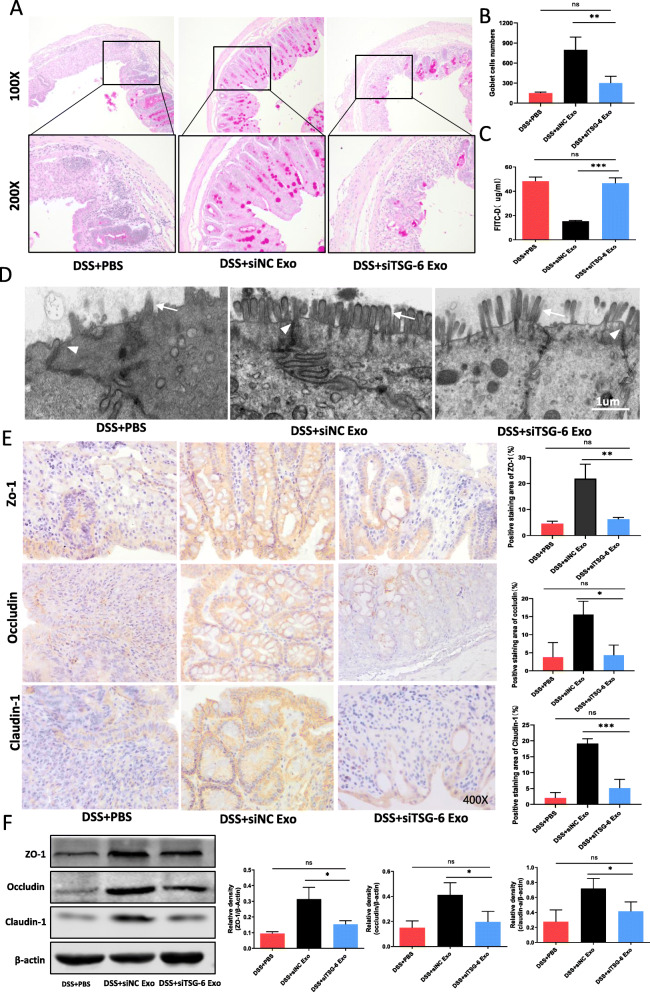
MSCs-Exo repaired colonic mucosal barrier via TSG-6 in colitis mice. **a** The number of goblet cells was significantly reduced in mice injected with siTSG-6 Exo compared to the DSS + siNC Exo group. **b** The histogram representing the number of goblet cells. **c** The histogram showing the concentration of FITC-D in the serum in each group of mice. **d** TEM images showing the ultrastructural morphology of tight junctions (white arrows) and microvilli (white arrowheads). The damage of tight junction and microvilli structure in the DSS + siTSG-6 Exo group were more serious than that in the DSS + siNC Exo group. Scale bar, 1 μm. **e** Immunohistochemistry analysis showing the expression levels of the tight junction proteins ZO-1, Occludin, and Claudin-1 in the intestinal epithelium. Images were taken at × 400 magnification. **f** Quantitative analysis of the expression of tight junction markers ZO-1, Occludin, and Claudin-1 in colonic mucosa by Western blot. Data are presented as mean ± SD. *P < 0.05, **P < 0.01, ***P < 0.001, and ns indicates *P* > 0.05

The above results confirmed that the anti-colitis effect of exosomes was mediated by TSG-6. However, it is still unclear whether MSCs-Exo exert its role of colonic mucosal barrier protection through TSG-6. As presented in Fig. 9a and b, the number of goblet cells in the DSS + siTSG-6 Exo group was significantly lower compared to the DSS + siNC Exo group. The intestinal barrier permeability of the mice treated with siTSG-6 Exo was higher than that with siNC Exo treatment, and no significant difference was observed from the DSS + PBS group (Fig. 9c). The damage of tight junction and microvilli structure in the DSS + siTSG-6 Exo group was more serious than that in the DSS + siNC Exo group (Fig. 9d). To explore the effect of MSCs-Exo on the colonic mucosa integrity after TSG-6 knockdown, immunohistochemical staining, and western blot were carried out to analyze the expression levels of TJ protein ZO-1, Occludin, and Claudin-1 in the intestinal epithelium of colitis mice. Results showed that siTSG-6 Exo treatment downregulated of ZO-1, Occludin, and Claudin-1 when compared to the DSS + siNC Exo group. There was no significant difference in other tight junction proteins between the two groups (Fig. 9e, f).

### Downregulation of TSG-6 blocked the regulation capacity of MSCs-Exo in the differentiation of CD4^+^ T cell treatment

**Fig. 10:**
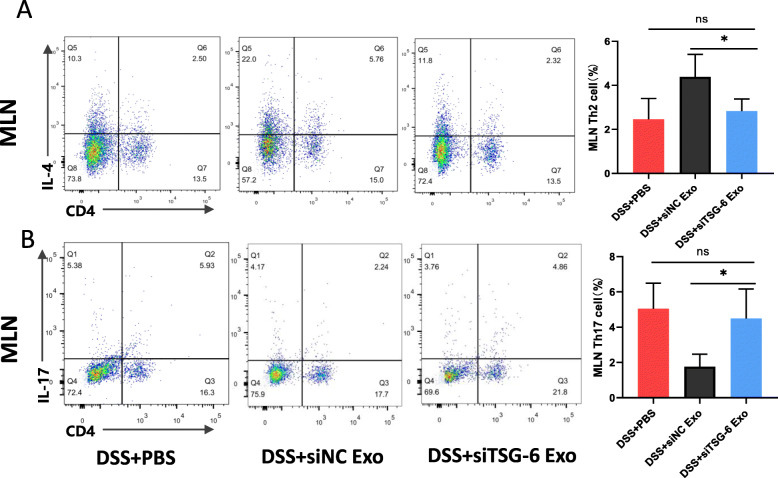
The proportion of Th2 cells and Th17 cells in MLN after treatment with TSG-6 siRNA knockdown MSCs-Exo (siTSG-6 Exo). Flow cytometry plots and graph analysis of Th2 cells (**a**) and Th17 cells (**b**) in the MLN from the DSS + PBS, DSS + siNC Exo and DSS + siTSG-6 Exo groups. Data are presented as mean ± SD; n = 4 per group. *P < 0.05 and ns indicates *P* > 0.05

To further clarify whether the improved balance between Th2 and Th17 cells after MSCs-Exo administration was TSG-6-dependent, MLN cells isolated from the DSS + siTSG-6 Exo and DSS + siNC Exo groups were subjected to flow cytometry analysis (Fig. 10a, b). The percentage of Th2 and Th17 cells displayed no significant difference between the DSS + PBS group and DSS + siTSG-6 Exo groups. However, increased proportion of Th2 cells and reduced proportion of Th17 cells were observed in siNC Exo-treated MLN.

### Injection of exogenous TSG-6 demonstrated a protective effect against colitis

**Fig. 11:**
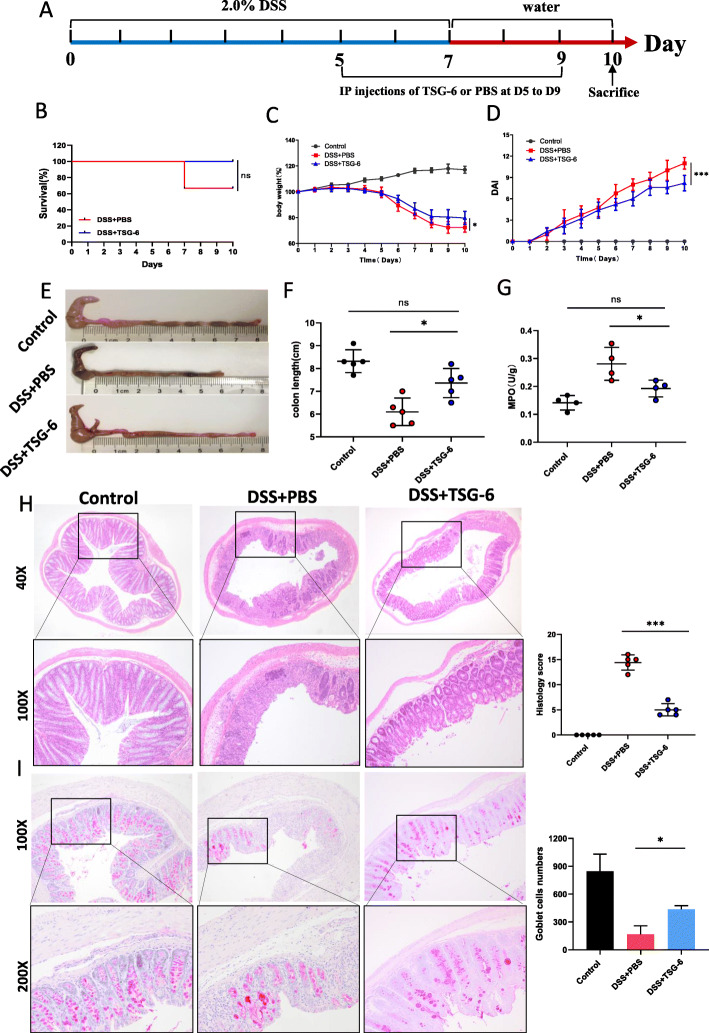
Injections of exogenous TSG-6 exerted a protective effect against colitis. **a** Schematic representation of experimental design. Recombinant human TSG-6 was injected intraperitoneally at 4 μg per mouse from day 5 to day 9. **b** Survival rate of mice in the DSS+ PBS and DSS + TSG-6 groups (n = 5–6 per group). Body weight (**c**), DAI (**d**), colonic macroscopic images (**e**), colon length (**f**), MPO (**g**), H&E staining and histological score (**h**) in the control, DSS + PBS, DSS + TSG-6 groups. **i** Quantitative analysis of PAS-positive goblet cells. Data are presented as mean ± SD. *P < 0.05, ***P < 0.001 and ns indicates *P* > 0.05

To further determine whether TSG-6 is a key mediator to relieve colon inflammation, TSG-6 was injected into DSS-induced colitis mice from day 5 to day 9 and its therapeutic effect was evaluated daily (Fig. 11a). The survival rate of colitis mice treated with exogenous TSG-6 was higher compared to the PBS treatment group; however, the difference was not statistically significant (Fig. 11b). TSG-6 also improved colon inflammation, which was reflected in body weight, DAI, length of colon, MPO activity, and histological changes in colon (Fig. 11c–h and Fig.S4d). In addition, TSG-6 also significantly increased the number of colonic mucosa goblet cells compared to the DSS + PBS group (Fig. 11i).

## Discussion

In this study, we investigated the effect of MSCs-Exo on colon inflammation. According to our experimental data, MSCs-Exo has significant therapeutic effects on both DSS- and TNBS-induced colitis models. Based on the ability of hUC-MSC in tissue repair, we explored the protective effect of MSCs-Exo on intestinal mucosal barrier. In addition, we found that the administration of MSCs-Exo affects intestinal immune response. MSCs-Exo treatment enhances the immune response of Th2 cells in MLN and reduced the immune response of Th17 cells. Next, we found that TSG-6 is detected in MSCs-Exo, and the therapeutic effect of MSCs-Exo is TSG-6-dependent. Our findings indicated that MSCs-Exo is a promising candidate for IBD treatment that may protect the intestinal barrier and modulate the immune response through TSG-6.

MSC are considered as a potential therapy for IBD and other autoimmune diseases [26]. Previously, our study found that injection of stem cells can improve colitis and its associated complications in mice [27]. Accumulating studies also revealed that the effects of anti-inflammatory and immunosuppression were mainly contributed by exosome secretion [28, 29]. Currently, various animal experiments have shown that MSC-derived exosomes exert therapeutic effects in treating colitis [13, 30], but only a single animal colitis model was used in most of these studies. The application of two different colitis models in our study reassured the anti-colitis effect of MSCs-Exo and suggested that the effect was strain-independent.

MSCs-Exo have been reported to stimulate the regeneration of epithelial cells in vitro [30], but its protective effect on the intestinal mucosal barrier in vivo remains unclear. The intestinal mucosal barrier includes mechanical, chemical, immune, and biological barrier. The intestinal barrier is a defense system against pathogen invasion, and intestinal barrier dysfunction contributes to IBD. The mechanical barrier is composed of TJs from the intestinal epithelial cells (IECs) and the mucus layer [31]. Mucus is produced and secreted by goblet cells in the IECs and contributes to IEC protection. We found that after DSS or TNBS administration, the number of goblet cells in the colonic mucosa is significantly reduced, and MSCs-Exo injection significantly rescues the goblet cell population. Our previous study demonstrated that ZO-1, Occludin, and Claudin-1 are the key members in the TJ protein family, which are pivotal for maintaining the function and integrity of the intestinal barrier [32]. Encouragingly, intraperitoneal injection of MSCs-Exo increases the expression of ZO-1, Occludin, and Claudin-1 in colon tissue and alleviates the disruption of the intestinal barrier. This phenomenon can also be observed directly under TEM. In addition, disruption of the intestinal barrier leads to increased intestinal mucosal permeability and promotes pathogen translocation [33]. Our results indicated that exosomes significantly reduce the permeability of the intestinal mucosa in colitis mice. These results fully substantiate the repairing effect of MSCs-Exo on intestinal damage.

As a pivotal line of defense against external antigens, the intestinal barrier interacts with the surrounding environment and immune cells [34]. When the barrier is destroyed, the paracellular permeability increases, leading to activation of immune cells [35]. MSC exert immunomodulatory effects on IBD [4, 36], so we examined whether exosomes secreted by MSC also have the immunoregulatory functions. We focused on evaluating the effect of MSCs-Exo on the immune response of the intestinal mucosa and found that MSCs-Exo treatment increases Th2 cell response while it inhibits Th17 cell response in acute colitis. CD4^+^ T cells are the key to mediate host protection and maintain immune homeostasis [37]. The imbalance between CD4^+^ T cell subsets, especially Th2 and Th17 cells, is the main factor driving IBD [37]. Recent studies showed that MSC ameliorate colitis by downregulating Th1 and Th17-mediated responses, while upregulating Th2 and Treg-mediated responses [38, 39]. We found that MSC-derived exosomes have the same immune regulation effect as MSC, which further verify the therapeutic effect of MSC in IBD. Unexpectedly, there is no significant difference in the proportion of Treg cells after the induction of colitis and MSCs-Exo administration. We speculate that it may be related to the acute colitis model in this study. During the acute colitis phase, the proportion of Treg cells in the lamina propria of the colon mucosa or MLN were not altered compared to the healthy mice [40]. Moreover, Th2 cells are involved in the transformation of activated T cells to an immunosuppressive phenotype in the acute inflammatory stage [40]. Th2 cells are important factors in maintaining the integrity of the intestinal mucosal barrier. When epithelial cells are damaged, Th2 cells response are initiated and Th2-related cytokines are secreted to facilitate tissue repair [41]. Th2-related cytokines, IL-4 and IL-13, are the main mediators of goblet-cell-produced mucus. Exogenous supplementation of IL-25 upregulates the expressions of IL-4 and IL-13 in the intestine, thereby promoting mucus secretion from goblet cells to restore mucosal barrier function [42]. In this study, MSCs-Exo treatment increases the proportion of Th2 cells and upregulates the expression of IL-4 in intestinal mucosa, which is consistent with previous literature.

TSG-6 is a secreted protein that exerts anti-inflammatory and tissue-protective properties [43, 44]. After receiving inflammatory signal, the corresponding cell releases TSG-6 to the injury site to suppress immune response and repair damaged tissue [45, 46]. It has been found in many animal models that the biological functions of stem cells are mainly mediated by TSG-6 [47–49], including IBD animal models [6, 19, 50]. To further explore whether TSG-6 mediates the effect of MSCs-Exo in the IBD treatment, we compared the expression levels of TSG-6 in the mice colon tissue treated with MSCs-Exo or PBS. Results showed that the expression of TSG-6 in mice injected with exosomes is significantly upregulated. Recent studies have revealed that extracellular vesicles from canine adipose tissue-derived MSC increase the polarization of M2 macrophages and Tregs through TSG-6 in vitro [6, 51]. Based on this, we proposed that MSCs-Exo may exert functions of immunosuppression and intestinal barrier repairing through TSG-6 in IBD. To test the hypothesis, we knocked down TSG-6 in MSCs-Exo with siRNA. Results indicated that MSCs-Exo functions to protect the intestinal barrier and regulate the diminished immune response. Moreover, exogenous supplementation of rhTSG-6 also mitigated IBD in mice.

Most of the current research regarding the MSCs-Exo-mediated IBD treatment focuses on immunoregulatory potential. However, the mechanism of MSCs-Exo on the intestinal barrier function remains unclear. Our study expands the understanding of the mechanism of MSCs-Exo in IBD treatment and provides an important step for the preclinical research of cell-free IBD therapy. Nevertheless, future studies will aim at exploring the downstream signaling pathway to repair mucosal barriers via MSCs-Exo-mediated TSG-6. Based on above experimental results, TSG-6 is one of the important mediators of therapeutic actions of MSCs-Exo, whether other proteins or non-coding RNAs from MSCs-Exo have an effect on IBD needs to be further investigated.

In conclusion, this study demonstrated that intraperitoneal injection of MSCs-Exo alleviates gut inflammation mainly by repairing intestinal mucosal barrier and maintaining immune balance. MSCs-Exo increase the expression of TSG-6 in the colon tissue of colitis mice after injection. After knocking down TSG-6, the protective effects of MSCs-Exo against colitis are significantly weakened. Therefore, the effects of MSCs-Exo on mucosal barrier maintenance and immune regulation might be partially mediated by TSG-6. This study indicated that MSCs-Exo may be a novel therapeutic approach for IBD treatment.

## Data Availability

The datasets used and/or analyzed during the current study are available from the corresponding author on reasonable request.

## Abbreviations

MSC: Mesenchymal stem cells
IBD: Inflammatory bowel disease
TSG-6: Tumor necrosis factor-α stimulated gene 6
MSCs-Exo: Exosomes derived from mesenchymal stem cells
DSS: Dextran sulfate sodium
TNBS: 2,4,6-Trinitrobenzenesulfonic acid
TJ: Tight junctions
MLN: Mesenteric lymph nodes
TEM: Transmission electron microscopy
NTA: Nanoparticle tracking analysis
siTSG-6 Exo: TSG-6 knockdown exosomes
siNC Exo: Negative control exosomes
PVDF: Polyvinylidene difluoride
H&E: Hematoxylin and eosin
